# Commonalities between Copper Neurotoxicity and Alzheimer’s Disease

**DOI:** 10.3390/toxics9010004

**Published:** 2021-01-07

**Authors:** Roshni Patel, Michael Aschner

**Affiliations:** Department of Molecular Pharmacology, Albert Einstein College of Medicine, 1300 Morris Park Avenue, Bronx, NY 10461, USA; roshnipatel6511@gmail.com

**Keywords:** Alzheimer’s disease, copper

## Abstract

Alzheimer’s disease, a highly prevalent form of dementia, targets neuron function beginning from the hippocampal region and expanding outwards. Alzheimer’s disease is caused by elevated levels of heavy metals, such as lead, zinc, and copper. Copper is found in many areas of daily life, raising a concern as to how this metal and Alzheimer’s disease are related. Previous studies have not identified the common pathways between excess copper and Alzheimer’s disease etiology. Our review corroborates that both copper and Alzheimer’s disease target the hippocampus, cerebral cortex, cerebellum, and brainstem, affecting motor skills and critical thinking. Additionally, Aβ plaque formation was analyzed beginning from synthesis at the APP parent protein site until Aβ plaque formation was completed. Structural changes were also noted. Further analysis revealed a relationship between amyloid-beta plaques and copper ion concentration. As copper ion levels increased, it bound to the Aβ monomer, expediting the plaque formation process, and furthering neurodegeneration. These conclusions can be utilized in the medical community to further research on the etiology of Alzheimer’s disease and its relationships to copper and other metal-induced neurotoxicity.

## 1. Introduction

### 1.1. Alzheimer’s Disease

Alzheimer’s disease is a progressive neurodegenerative disorder that targets neuron communication and can result in loss of cell function or cell death [1,2,3]. This is due to the buildup of abnormally structured proteins called amyloid plaques and neurofibrillary tangles between neurons, essentially blocking communication [3]. Nerve cell death consequently results in loss of brain tissue. Alzheimer’s disease initially disrupts bodily communication, metabolism, and repair processes [3]. As its effects advance, the patient may experience memory loss as well as changes in language, reasoning, and social behavior [3]. Alzheimer’s disease is also a fatal form of dementia, and, according to a 2020 study, is the sixth-leading cause of death in the United States [4].

It is estimated that one in 10 Americans age 65 or older, about 5.8 million people, suffer from Alzheimer’s disease [5]. Women show a greater prevalence of Alzheimer’s disease with 3.5 million cases, about two-thirds of Americans with Alzheimer’s disease, while men show a prevalence of 2.1 million [6]. Between 2000 and 2017, the death rate due to Alzheimer’s disease increased by 145% with 121,404 cases reporting Alzheimer’s disease as the cause of death in 2017 [6]. This number is expected to rise in the coming years as it is estimated that the prevalence of Alzheimer’s disease in the United States will continue to grow to 13.8 million [6].

Alzheimer’s disease proves to be a major social and financial burden on the families of those affected. It is estimated that 83 percent of unpaid aid, referred to as informal care, provided to those with Alzheimer’s comes from family members, friends, or unpaid caregivers [7]. One study estimated that in 2018, over 18.5 billion hours of informal care were provided to those with Alzheimer’s disease and like dementias, equating to $233.9 billion [6]. Lifetime costs of someone with Alzheimer’s disease are estimated at $350,174 where 70 percent of these costs are associated with family care [8]. This poses a significant problem as 41 percent of informal caregivers have a household income of less than $50,000, putting an outstanding strain on family members, especially since many of those placed in these situations often believe they had no choice in taking on such a role [6].

### 1.2. Copper

Copper, an essential trace element found in the brain, liver, and kidneys, enables the body to form red blood cells, maintain bone health, and can help prevent cardiovascular disease and osteoporosis. Copper is also a key element in maintaining lung function as it plays a vital role in metabolic processes such as cellular respiration [9]. Copper stored in the human body can be used for protein and energy production [10,11]. For adults, healthy copper levels range between 50 and 80 mg; levels exceeding this range are considered toxic and can lead to a buildup of copper in the kidneys, brain, and eyes. This causes a burden on the body as it may result in possible liver cell death, permanent nerve damage, oxidative stress, and reduced cell proliferation [10,12]. A lethal dose of copper ranges from 10 to 20 g [12]. Common signs of copper toxicity include, but are not limited to, headaches, bloody vomit, diarrhea, and black stools.

In the bloodstream, copper exists in two forms: 85 to 90 percent of copper found in the blood is bound to ceruloplasmin, a protein that plays a role in iron metabolism; the remaining 10 to 15 percent is free-floating copper, sometimes loosely bonded to other molecules [12,13]. Copper toxicity may occur as a result of various exposures. Common means of excess copper exposure occur from consumption of acidic foods cooked in uncoated copper cookware, exposure to excess copper in drinking water, breathing air or dust containing copper, as well as other environmental sources [12,14]. Other instances include copper salt topical creams for burn treatment, as well as in farming as a pesticide, and the leather industry [12].

Several genetic disorders are associated with copper related diseases. Examples of such illnesses include Wilson’s disease and Menkes disease. Wilson’s disease, a genetic disorder where the body is unable to filter out excess copper and therefore builds up in body tissue, occurs due to mutations in the ATP7B gene [13]. ATP7B codes for the protein ATPase 2, a copper-transporter found in the liver and brain [13,15]. This mutation can result in hepatic toxicity as well as adverse effects on the central nervous system, disrupting homeostatic bodily functions [13]. Similarly, Menkes disease, an X-linked recessive disorder, is a result of mutation in the ATP7A gene; the purpose of ATP7A is to code for copper regulation and copper absorption from food [15]. Symptoms of Menke’s induced copper deficiency include hair loss, slow growth and development, and neurological effects [15].

## 2. Methodology

**Figure 1:**
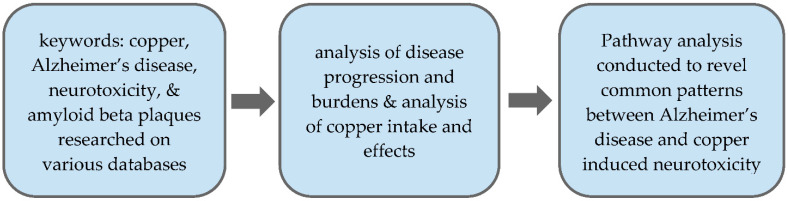
Methodology overview.

This study aimed to clarify the relationship between copper toxicity and Alzheimer’s disease, more specifically the effect of copper on neurological pathways and amyloid-beta plaque formation. Such an association has been previously studied by various researchers; therefore, this study utilized a systematic review approach of published literature to synthesize the known data and information regarding this relationship (See Figure 1). Data sets such as PubMed, Science Direct, and Google Scholar were utilized to collect journals, publications, and review journals relating to the topic. Journals were chosen using keywords: copper, Alzheimer’s disease, neurotoxicity, and amyloid beta plaques. These data sets were sourced using Zotero, and online reference manager. These journals were reviewed and trends and patterns regarding copper toxicity and its relationship with Alzheimer’s disease were analyzed.

To begin the data collection process, preliminary research was conducted to gain an overarching understanding of the topic. Such research included analyzing the progression and development of Alzheimer’s disease and its effects on a patient and those around them. Social and financial burdens were also analyzed to develop the societal impacts of Alzheimer’s disease. Similarly, journals pertaining to copper toxicity and its effects on the brain were analyzed to understand the common causes of copper toxicity, the prevalence of copper in everyday life, and common forms of entry into the human body.

Once comprehensive background knowledge of the individual research subjects was obtained, trends and patterns relating the two were sought and noted. Firstly, patterns in the pathways of both copper and Alzheimer’s disease effects were noted in order to analyze if a correlation could be derived between the two. Patterns were analyzed in terms of the route copper and Alzheimer’s disease took in the brain; pathways were named in terms of the section of the brain that was affected, such as cerebral cortex, cerebellum, and brainstem. As more trends arose, they were subsequently categorized into their respective clusters, such as common pathways of copper and Alzheimer’s disease, as well as the relationship between Aβ plaques and copper binding sites. After the organization of the detailed clusters was analyzed and categorized respectively, sections were created to study the beta-amyloid plaque and copper relationship. This area included the analysis of plaque formation from protein strand to tangle as well as how shape affects the purpose of the plaque. Then, copper’s relationship to the development of these plaques was introduced and the two were analyzed in concurrence to reveal significant findings such as coppers role in plaque development as well as how different copper concentrations affect plaque growth.

## 3. Copper Pathways to the Brain

### 3.1. Role of Copper in the Human Body

Copper is an element essential to the homeostasis of the human body due to its role in energy production, iron metabolism, neuropeptide activation, and connective tissue and neurotransmitter synthesis [16]. Copper’s most crucial property however is its role in cuproenzyme ceruloplasmin [17]. Ceruloplasmin is the major copper-carrying protein in the blood, accounting for over 95% of copper transport in human plasma [16,17]. Studies have also found that copper plays a crucial role in the formation of red blood cells, immune system function, brain development, gene expression, and other physiologic processes [16]. Through the use of ceruloplasmin transport, copper is able to reach the brain, which uses a significant portion of copper found in the body for brain development and regulation of the nervous system [13,16,17]. Copper is also significantly used in the liver to convert iron into its ferric form as well as absorb iron into the gut [13].

### 3.2. Common Forms of Copper Intake

Copper toxicity is an associated result of exposure to copper contaminated sources. Common exposure sites include water supplies, copper pipes, uncoated copper cookware, birth control, dietary supplements, food, and fungicides. Water supplies are often contaminated by farm operations and other industrial waste practices that become runoff and enter reservoirs and public wells [18]. This waster is then transported to nearby populations for consumption and other daily activities. Such an instance was accounted for in a 2016 journal highlighting the widespread water contamination of heavy metals, specifically copper and lead, in New South Wales, Australia [18]. This study analyzed tap water from 212 different homes in the region; of these samples, almost 100 percent tested positive for copper, with 5 percent testing positive for excess copper [18]. For the houses testing within the normal copper range, it was also found that drinking water contributed to 6 to 13 percent of the average daily intake of copper [18]. A high copper positive percentage was expected since many of these homes utilize copper pipes and fittings for water supply [18]. Subsequently, copper pipes are a great cause of concern regarding copper contamination as acidic water may cause erosion of the pipe or fitting, releasing copper particles directly into the water [18]. Therefore, this study concluded there is a great concern for copper levels due to their frequent occurrence at high concentrations, as this water is then used for drinking, washing of cookware, as well as for bathing, and personal hygiene, creating various opportunities for direct ingestion of copper contaminated water, increasing the prevalence of copper toxicity in the region [18]. Other sources of copper contamination include improperly coated copper cookware, such as pots and pans. If not coated with another non-reactive metal, copper from the cookware can enter food and therefore the human body. As copper levels in the body increase, excess copper begins to pool in tissues, leading to copper toxicity. Similar to copper pipes, unprotected copper cookware can corrode in the presence of acidic foods, resulting in another means of entry into the human body [12]. Copper toxicity can also be caused through the use of birth control, as it can raise copper levels, which also destroys Vitamin C, another essential nutrient for the human body’s optimal function [19]. Dietary supplements and other foods can also be a source of excess copper. The recommended daily dose of copper for those aged 19 and older is 900 mcg, with the upper limit at 10,000 mcg [13]. Overconsumption of copper supplements or foods high in copper content, such as legumes, mushrooms, chocolate, liver, and nuts and seeds, can result in copper toxicity when not consumed in moderation [13]. Copper sulfate, an inorganic copper and sulfur compound used as a fungicide and algaecide in swimming pools, creates a toxic environment for microorganisms that kill small plants and animals by shocking roots and causing copper toxicity, respectively [20]. Due to its multiple points of entry into the human body, copper contamination is a growing concern, especially considering the large quantities in which it is found in the activities of daily life.

### 3.3. Alzheimer’s Disease and Copper Pathways

The adult human brain contains approximately 100 billion neurons, directly correlating with brain mass. As healthy aging occurs, neurogenesis, the process of regeneration by neural plasticity, slows down, and neuron proliferation rate decreases [21]. Dementias, such as Alzheimer’s, can speed up neurodegenerative processes, causing memory loss, progressing to the point where the patient is no longer capable of independently performing activities of daily living. Referred to as cerebral atrophy, the loss of neurons decreases brain mass overall or in specific areas [22]. Alzheimer’s disease begins by targeting the hippocampus and entorhinal cortex located in the temporal lobe [3,23]. The temporal lobe is responsible for the connection of the network responsible for memory, navigation, and the perception of time [23]. The targeting of this region decreases the number of transmitters in the temporal lobe, causing neuron death and therefore size reduction, which can be seen on MRI scans of the brain [23]. A 2020 study found that the temporal lobe of those with Alzheimer’s disease decreases at a rate of 15.1 percent per year whereas the normal neurodegeneration rate is a significantly lower 1.5 percent [24]. The study concluded that cognitive decline and subsequent memory loss are a direct result of temporal lobe atrophy [24]. As the disease progresses, the cerebral cortex also becomes impaired [3,25]. The cerebral cortex is the outer layer of neural tissue located at the front of the head, covering the outer portion of the cerebrum [26]. The cerebrum controls language, reasoning, social behaviors, emotion, muscle movement, hearing, vision, and other sensory controls [3,26]. Alzheimer’s disease can also be characterized by neural mass loss and astrocytosis in the cerebral cortex [3,25]. A 2018 study found that about 25 percent of individuals who die by the age of 75 presented with substantial cerebral lesions resembling those of Alzheimer’s disease [25]. This study concluded that the identification of cellular brain structure is essential to understanding neurodegenerative disease progression [25]. The order of progression for Alzheimer’s disease begins in the hippocampus and expands outward to the temporal lobes, frontal lobes and prefrontal cortex, parietal lobes, occipital lobes, cerebellum, and finally the brainstem [27]. Once the disease reaches the brain stem, autonomous functions of the body will cease, ultimately proving fatal [27].

Copper toxicity results in the pooling of copper in different tissues of the body [10,12,28]. Prominent areas of copper pooling include the liver, brain, and eyes [12]. A 2013 study of male Wistar rats conducted by Pal et al. found copper toxicity effects on the rat brain include swelling and increased number of astrocytes, star-shaped glial cells, and copper deposition in the choroid plexus, which is located in close contact with the cerebral cortex [29,30]. The study concluded that copper toxicity in male Wistar rats causes impaired spatial memory and neuromuscular coordination, swelling of astrocytes, copper deposition in the choroid plexus, neuronal degeneration, and augmented levels of copper in the hippocampus [29]. This is a significant finding due to the implications of these symptoms; impaired spatial memory and neuromuscular coordination can result in difficulty walking and navigating, swelling of astrocytes can result in brain edema and fulminant hepatic failure, impairment of the choroid plexus can cause variations in the development of cerebrospinal fluid possibly leading to overproduction causing pressure in the brain, neuronal degeneration can lead to neuron death, and augmented levels on copper in the hippocampus can lead to various dementias [31,32,33,34]. Dementias may be a result of the various plaques copper can induce, the most prevalent of which are amyloid-beta plaques associated with Alzheimer’s disease [35]. Another 2018 study, conducted by Kardos et al., found copper levels to be significantly higher in the cerebellum, choroid plexus, ventricle system, and substantia nigra, a region of the midbrain [36]. This study also found that free excess copper often pools in the soma of cerebellar granule and cortical pyramidal neurons, as well as the hippocampus and spinal cord [36]. One 2012 study analyzed possible reasons for high copper levels in the brain and theorized that a high-fat diet caused an increase in copper levels, which they theorized was also correlated to an increased risk of Alzheimer’s disease [37]. The study found that the highest 20 percent of those with copper intake lost cognition at six times the rate of groups with lower copper consumption if they also had a high-fat diet [37]. The study hypothesized that the ingestion of free-floating copper from sources such as drinking water and copper supplements played a major factor in the onset of Alzheimer’s as it caused high levels of copper pooling in the brain [37]. This study concluded that there is a strong correlation between copper levels and the prevalence of Alzheimer’s disease [37]. It is believed this correlation exists due to the commonalities between affected areas by Alzheimer’s disease and copper toxicity [36,37]. Such common areas include the hippocampus, cerebral cortex, cerebellum, and brainstem, which affect memory, information processing, motor skills, and regulation of autonomous functions, respectively [27,36,37]. This supports the theory that copper may play a role in the onset of Alzheimer’s disease as these regions are also the pathway of the general progression of the disease [27].

## 4. Beta-Amyloid and Copper Relationship

### 4.1. Beta-Amyloid Plaques

Alois Alzheimer characterized Alzheimer’s disease as an elevated number of plaques in the brain in the early twentieth century [38]. Then, in the 1980s, it was found that these plaques consist of amyloid-beta (Aβ) peptides, protein fragments from the amyloid precursor protein (APP) known to play an important role in synaptic physiology by regulating synaptic vesicle release and scaling [38,39]. This was an important discovery as the amyloid precursor protein had been known to regulate cell growth, motility, neurite outgrowth, and cell survival [39]. Aβ peptides are also naturally produced in the brain and normally exist in healthy levels in the cerebrospinal fluid and serum [40]. As research in the field progressed, John Hardy and Gerald Higgins proposed the Amyloid Cascade hypothesis in 1992 [41].

The Amyloid Cascade hypothesis postulates that as proteostasis changes due to aging, the amyloid precursor protein is broken down to form Aβ, which results in an abnormal accumulation of amyloid-beta plaques in various regions of the brain [35,42]. This hypothesis was significant since the formation of the Aβ monomers could cause the deposition of extracellular fibrils, resulting in neuron death and the formation of senile plaques [35]. A 2014 study on the Amyloid Cascade hypothesis, however, found that low doses of Aβ are stimulatory to the brain whereas high doses were inhibitory [38]. Since advanced medicine has been unable to find a cure for Alzheimer’s disease based on this hypothesis, new avenues of approach are being investigated, leading to the development of new lines of reasoning [43]. One such new hypothesis that challenges the widely accepted Amyloid Cascade hypothesis is the Beta-Amyloid Dysfunction (BAD) hypothesis proposed by Heinz Hillen in 2019 [43]. The BAD hypothesis differs from the Amyloid Cascade hypothesis by building on the homeostasis of the Aβ peptide in the synaptic vesicle cycle [43]. Further, the BAD hypothesis accounts for the physiological Aβ monomer, the pathophysiology of Aβ deposits, and reduced Aβ cerebrospinal fluid levels in Alzheimer’s disease whereas the Amyloid Cascade hypothesis does not [43]. Other hypotheses that were proposed but are not widely accepted include the infection hypothesis and vascular hypothesis [35].

The amyloid-beta buildup can cause multiple health concerns and detrimentally impact patient health. When Aβ plaques accumulate outside nerve cells, neural synapses are blocked and action potentials, or electrical impulses in the brain, are blocked, hindering communication and resulting in neurodegeneration [44,45,46]. This can cause a variety of illnesses, such as Alzheimer’s disease, Parkinson’s disease, Huntington’s disease, type 2 diabetes, amyotrophic lateral sclerosis, secondary amyloidosis, and prion diseases, among others [47]. In Alzheimer’s disease specifically, these Aβ plaques can also result in neurofibrillary tangles, further hindering neuron communication [35]. Mutations in the previously described APP protein cause the development of amyloid-beta plaques due to improper folding of the peptide [48,49]. A 2017 study by Han et al. examined how Aβ-42 induces neural apoptosis; the study concluded that Aβ-42 targets the mitochondria of the neuron, causing neurodegeneration [50].

The human body has some protection against the buildup of Aβ monomers in the form of brain macrophages which can engulf and destroy the peptide, called microglia [35]. However, the aging process results in a decreased amount of these macrophages, allowing for unchecked Aβ buildup [35]. Unregulated buildup induces an inflammatory response, causing neuroinflammation and neuron death [35]. This response works in conjunction with Tau proteins which are also stimulated by the inflammatory response, creating neurofibrillary tangles that restrict neuron function and lead to neurodegeneration [35].

**Figure 2:**
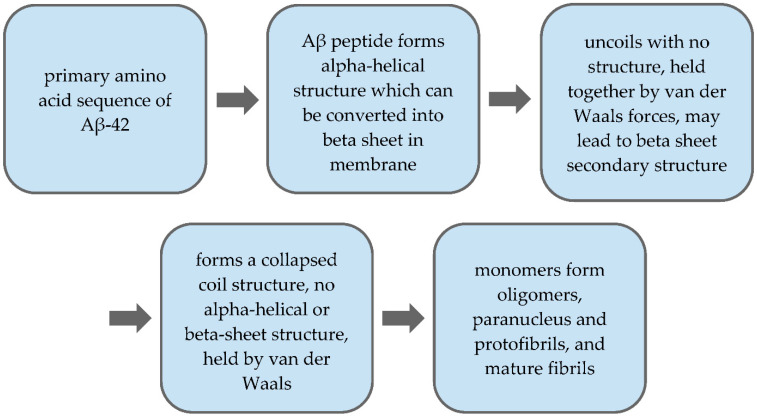
Structural process of Aβ-42 monomer to Aβ fibrils.

Amyloid-beta peptides are created through the incorrect folding of proteins, resulting in amyloidosis [51]. Protease, an enzyme that breaks down large proteins, recognizes these abnormalities and discards them [51]. However, sometimes the amyloid to protease ratio is imbalanced, with more amyloids than the enzymes can handle, resulting in a buildup [51]. Other instances resulting in buildup are caused by the formation of a rigid structure from the amyloid mass, which exceeds the protease’s capabilities [51]. When this mass is excreted out of the cell, it folds to create a beta-sheet, which can go on to wreak damage on the area, as seen in the case of Alzheimer’s disease [49]. The Aβ peptide consists of 37 to 49 amino acids that stem from the previously defined APP protein [48]. These monomers can aggregate into various structures, including oligomers, protofibrils, and amyloid fibrils, which are usually larger than the latter two and insoluble [48]. Due to their larger size and insolubility, these fibrils can rearrange themselves into clumps, forming Aβ plaques [48]. Therefore, the APP’s amyloid fibril form is said to be a primary component of amyloid plaques found in the brain during Alzheimer’s disease [48,49]. These plaques are most commonly found in the neocortex, a part of the cerebral cortex associated with sight and hearing [48]. Figure 2 derived from the findings of Chen et al. provides more information into the process by which Aβ fibrils are created.

It is important to note that Aβ is thought to be intrinsically structured, and therefore is unable to be crystallized [48]. Therefore, scientists aim to optimize conditions to stabilize the Aβ peptide in order to study its properties [48]. The primary Aβ-42 amino acid sequence was discovered in 1984 from extracellular deposits and amyloid plaques and was found to range from 37 to 49 amino acids [48,49]. Early models of the Aβ peptide found it to fold into α-helical and β-sheet structures in membranous environments [48]. Portions of the peptide structure then uncoil and either form various or no structures, depending on their placement in the peptide chain; a helix to coil transformational transaction is also promoted [48]. The Aβ peptide collapses into nonstructural loops, strands, and turns when placed in water [48,49]. Although this chain no longer has a set structure, it is held together by van der Waals forces [48]. Beta secondary structures may be formed through fibrillization, and two helical regions connected by a β-turn are formed [48]. It is believed that this aggregation of Aβ peptides into Aβ fibrils is what causes the onset of neurotoxicity and dementias such as Alzheimer’s disease [48].

### 4.2. Copper Toxicity and Aβ Levels

**Figure 3:**
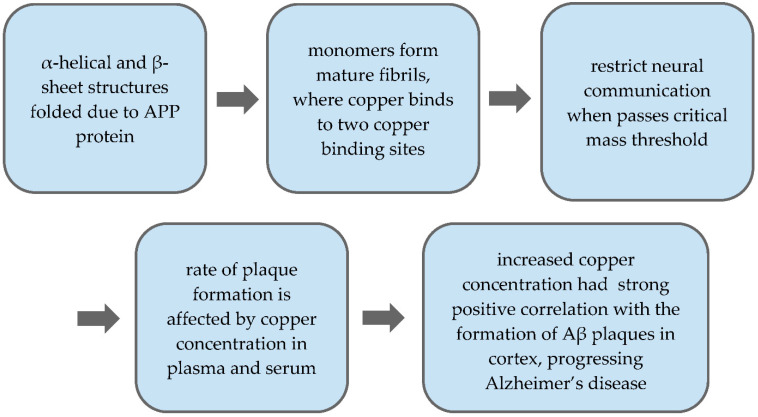
Process of APP protein to Aβ plaque formation in accordance with the effect of copper.

Alzheimer’s disease is characterized by neurodegeneration, elevated heavy metal concentrations in the brain, and amyloid-beta plaque buildup [40]. Some heavy metals that are believed to have a relation to the onset of Alzheimer’s disease include zinc, iron, and copper [40]. Of the three, copper is believed to have a more significant impact on the development of Alzheimer’s disease due to the amyloid-beta plaques two copper-binding sites, which can be used to promote reactive oxygen species (ROS) generations [1,40]. When bound to plaques, copper cycles between the +I and +II oxidative states, boosting ROS generation; ROS may damage lipids, RNA, DNA, or proteins, effectively advancing aging and possibly Alzheimer’s disease [52,53]. One 2018 study conducted by *Bagheri* et al. found that copper ions have a high affinity to the Aβ peptide copper-binding site, increasing the proportions of β-sheet and α-helix structures in Aβ aggregations, forming plaques [1]. This study also found that increased copper ion concentrations result in greater fibril formation, once again increasing Aβ plaque buildup [1]. This happens due to the β-amyloid’s ability to transform into β-sheet structures when bound to two copper ions, which not only stimulates the aforementioned β-sheet conformity but also aggregation and toxicity, as depicted in greater detail in Figure 3 [1]. In vitro studies performed in the early 2000s were able to confirm the formation of these structures in copper toxic Alzheimer’s patients [1].

Several factors are believed to play a role in the copper’s ability to bind to these Aβ aggregates, inducing a fibril formation process [54]. Such factors include pH of 7.0, increased concentrations of free copper in plasma, and serum copper concentrations, which can also cause the onset of a rare type of Alzheimer’s disease [1,54,55]. Furthermore, the study conducted by Bagheri et al. found a 1:1 ratio of free-floating copper in serum to Aβ aggregates, a significant advancement in the development in the comprehensive understanding of Alzheimer’s disease [1]. Over the course of its six meta-analyses, this study aimed to evaluate copper concentrations in AD in different biological matrices, this study concluded that free copper/copper ion, concentration in serum was significantly greater in patients who were diagnosed with Alzheimer’s disease than those who were considered healthy [1]. This conclusion helps develop past findings, such as the fact that Aβ plaques exhibit high copper concentrations among other trace metals, to understand the reasoning behind why such phenomena occur [1].

As previously described, this increase in copper leads to an increase in Aβ plaques through the bonding of free copper and β structures. This bonding causes the shape of the β sheet to change again, creating fibrils that can aggregate. These masses can then hinder the brain’s ability to successfully communicate information and allow the body to function. However, another 2016 study by Kitazawa et al. establishes an alternate line of reasoning for this process [56]. In this study, Kitazawa et al. propose that high copper ion concentrations in serum can trigger an inflammatory response in the brain [56]. This inflammatory response in turn obstructs the brain from breaking down Aβ aggregates, causing a buildup as seen in Alzheimer’s disease [56]. Essentially, this study focused on excess copper concentration and its effects on microglia, which were previously defined as the body’s natural way to break down and dispose of excess Aβ plaques before they pose a threat to the patient’s homeostatic processes [35,56]. This study found that excess copper concentrations correlated with a decrease in microglial activity as well as brain inflammation [35,56]. When operating in conjunction, these processes accelerate neurodegeneration and the formation of Aβ plaques, detrimentally affecting the patient’s brain twofold [35,56]. This study, like others cited herein, concluded that copper toxicity may trigger the progression of Alzheimer’s disease by targeting homeostatic processes [1,35,40,54,56].

## 5. Conclusions

This study sought to investigate the relationship between Alzheimer’s disease and copper toxicity. Initial research into the individual factors found Alzheimer’s disease to be one of the most prevalent forms of dementia, accounting for 5.8 million cases in the United States alone [5]. As there is no cure for this illness to date, the disease proves fatal [4,27]. Care for those who suffer from Alzheimer’s disease is extremely costly; those from lower-income demographics diagnosed with this illness face a significant financial strain, further establishing why a cure for this illness is needed [7]. Alzheimer’s disease targets neuron function through the formation of amyloid-beta plaques, which hinder the neuron’s ability to successfully transmit nerve impulses, causing neurodegeneration including cell death [3,57]. In turn, this loss of brain mass results in decreased cognitive and motor skills [24]. This study sought to understand the role of copper in the etiology of Alzheimer’s disease.

Alzheimer’s disease manifests in the inner portions of the brain, such as the hippocampus and temporal lobes, and extends outwards with the following order of progression: temporal lobe, frontal lobe, prefrontal cortex, parietal lobe, occipital lobe, cerebellum, and brainstem [27,36,37]. When analyzed alongside the regions targeted by Alzheimer’s disease, commonalities with copper toxicity, such as accumulation in hippocampus, cerebral cortex, cerebellum, and brainstem were found, concomitant with adverse effects on memory, information processing, motor skills, and regulation of autonomous functions [1,24].

We further reviewed the relationship between amyloid-beta plaques and copper. Firstly, we reviewed the structure and formation of Aβ beginning from its parent protein, APP [38,39]. From the APP protein, structures such as α-helical and β-sheets were addressed to determine the process by which such structures are folded [48]. Finally, the process from monomer to plaque, including aggregation, was addressed [48]. The effect of copper ions on these plaques was then reviewed, suggesting that excess copper, given its high affinity for Aβ plaques, can bind to the plaques at two distinct copper-binding sites [1,40,54]. When these plaques reach a critical mass and cluster between neurons, they restrict neuronal communication [1,54,56]. Factors that affect the rate of plaque formation include copper concentrations in plasma and serum [1,54,55]. Increased copper concentration had a strong positive correlation with the formation of Aβ plaques in the cortex, advancing the progression of Alzheimer’s disease [1,35,40,54,55,56].

These findings can be used in the medical field to derive treatment options and solutions to this ongoing problem. An additional timely study is to target the copper-binding site in the Aβ monomer to investigate how this change affects Aβ plaque generation. Another study should investigate if free-floating copper also plays a role in the rate at which Aβ monomers aggregate into Aβ plaques, as this would provide insight into how to directly combat the formation of plaques.

## Data Availability

Not applicable.
